# Reorienting the Way a Community Mental Health Service Responds to Suicide and Other Serious Incidents

**DOI:** 10.1007/s10597-026-01604-x

**Published:** 2026-03-21

**Authors:** Nandi Abdalla, Richard Whitehead, Liza Hopkins, Paul Denborough, Rachel Barbara-May, Michelle Kehoe

**Affiliations:** 1 Alfred Health, Melbourne, Australia; 2 Deakin University, Burwood, Australia; 3 Monash University, Melbourne, Australia

## Abstract

Suicide and other serious incidents involving users of mental health services can have a deep and lasting effect on staff members who have been involved in the person’s care. It is important for the service to ensure that staff can learn and improve from these incidents while also minimizing the negative impacts on workers. One service has developed an innovative approach to reviewing and responding to serious incidents, using a dialogically informed, learning circle approach, based on the principles of restorative justice. This approach seeks to support staff to manage workplace stress and distress, while also acknowledging the person involved in the incident as well as the rights and needs of the family members and friends. It is intended that such an approach, rather than blaming staff, seeks to support them and build a service in which it is safe to practice in a person-centred, non-defensive way. This paper reports on the experiences of staff within this service who have taken part in this serious incident review process, and provides support for the roll out of the model as well as highlighting some of the pitfalls and difficulties in implementing such a model within current service structure guidelines. The project found that having a process which was supporting and not punitive enabled staff to feel supported to remain in the workforce and to improve their practice within a supportive, learning-oriented organisation.

## Introduction

Suicide, death by misadventure and other serious incidents are a regular occurrence at mental health services (National Confidential Inquiry into Suicide and Safety in Mental Health, 2023). Beyond the impact on the community members and familial and social connections, suicides and other serious incidents can have a major impact on mental health workers (Haylor et al., 2024; Holden & Card, 2019; Lyra et al., 2021; Niishi et al., 2025; Sandford et al., 2020; Wilshke & Crepeau-Hobson, 2024). The way in which mental health services investigate and deal with such incidents is important for everyone involved, and steps need to be taken to ensure mental health services can learn and improve from these incidents while also minimizing the negative impacts on their workers.

Current models available for dealing with serious incidents in mental health services often rely upon processes such as root cause analysis (RCA) that seeks to find a linear root cause of the incident through analysing documentation such as case files and client notes (Deshpande et al., 2023). However, questions have been raised regarding the appropriateness of RCA in mental health services (Deshpande et al., 2023; Haylor et al., 2024; Vrklevski et al., 2018), as linear causality is rarely found (Deshpande et al., 2023; Vrklevski et al., 2018). Despite the rigorous nature of the RCA process, it does not directly address the negative impact on mental health staff, following a suicide or other serious incident. Thus, there is a need to investigate additional models of responding to serious incidents (Fröding et al., 2024; Hagley et al., 2019) where services can reflect and learn, while also providing support to all parties involved. The aim of the present study is to investigate the experience of mental health service staff taking part in a dialogically informed, non-punitive, person-centred process to investigate serious incidents known as the serious incident review panel (SIRP).

## Impact of Suicide on Mental Health Workers and Services

The impact of suicide on the community is widespread. Suicide remains the leading cause of death for young people aged 15–25 in Australia (Australian Institute of Health and Welfare, 2025). Beyond the impact on families (Kehoe et al., 2025), there is also a significant impact on the mental health workers who have been involved in the care of these young people (Haylor et al., 2024; National Confidential Inquiry into Suicide and Safety in Mental Health, 2023; Pratt & Jachna, 2015) and a flow on effect on the culture and practice within mental health care services (Rytterström et al., 2020).

A recent systematic review of the impact of patient suicide on mental health practitioners (Sandford et al., 2020) found mental health professionals affected by patient suicide experienced feelings of anger, sadness, shame and self-blame. Ruskin et al. (2004) reported that psychiatrists and psychiatric trainees felt shock and horror in response to their first patient suicide, and 20% met the criteria for PTSD following such incidents, with a further 22% meeting the criteria for acute stress disorder. Croft et al. (2023) also found some staff experienced flashbacks and hyper-arousal following the suicide of a patient. In some cases, the suicide of a patient was also associated with the increased suicidality of mental health care staff (Ruskin et al., 2004; Pratt & Jachna, 2015; Sandford et al., 2020).

The impact of patient suicide can extend beyond the personal effect on service providers and can affect how support is provided to others. Following a patient suicide, clinicians often experience greater professional self-doubt (Gulfi et al., 2010; Kelleher & Campbell, 2011; Sandford et al., 2020) and may engage in more risk averse and ‘defensive practice’ (Croft et al., 2023; Sandford et al., 2020). For example, following a patient suicide, studies show some clinicians are more likely to prescribe antidepressants and use hospital admissions as a way to detain patients and manage risk (Gulfi et al., 2010; Halligan & Corcoran, 2001; Sandford et al., 2020). Some clinicians even stated they were less likely to make connections with clients’ families following a previous patient suicide (Kelleher & Campbell, 2011), while others contemplate leaving their profession altogether (Sandford et al., 2020).

Acknowledging the impact of patient suicides on clinicians highlights an important issue for mental health services, which have both a moral obligation and a practical imperative to support staff experiencing the secondary impacts of suicide (Pratt & Jachna, 2015). By addressing the emotional toll on staff, services not only improve well-being and morale, but can ensure staff are not engaging in defensive practice or protecting themselves by working in a manner that neglects important relational and person-centred work (Sandford et al., 2020). Evidence suggests that the quality of support the clinicians receive after a serious incident such as suicide can mitigate the detrimental impact of the suicide (Croft et al., 2023; Pratt & Jachna, 2015; Sandford et al., 2020) as long as follow up support does not involve the attribution of blame (Kelleher & Campbell, 2011; Murphy et al., 2022). The concept of a no-blame approach to address issues lies at the heart of restorative justice (Kehoe et al., 2018). Restorative justice considers that when a harmful situation has occurred, it results in damaged relationships, and that those relationships need to be repaired (Ahmed & Braithwaite, 2012). The use of restorative justice offers a space to openly express concerns to maintain and strengthen relationships (Morrison et al., 2005), including those within the hierarchy of the organisation as well as between staff members and their clients and family members.

## How do Organisations Deal with Serious Incidents?

In many healthcare services there is an expectation that an investigation of all serious incidents will be carried out to identify any possible patient harm caused by the service, and to assess for any legal ramifications from the incident (Fröding et al., 2024). When investigating suicides, due to the varied presentations of suicidal people, and lack of clear linear cause and effect, finding a direct cause or fault with any one point of client contact can be challenging (Fröding et al., 2021, 2024; Haylor et al., 2024; Vrklevski et al., 2018). Failure to consider this nuance when reviewing suicides and other serious incidents can have adverse effects on individuals and hinder the growth and learning in services needed to improve patient safety outcomes (Nicolini et al., 2011).

## Root Cause Analysis

One of the current common methods of investigating adverse incidents in mental health services is through a root cause analysis (RCA; Vrklevski et al., 2018), with RCAs being mandatory in many health jurisdictions. RCAs have been praised for being a systematic process that provide a formalised acknowledgement of serious incidents (Haylor et al., 2024; Vrklevski et al., 2018) and, ideally, the findings of RCAs are utilised to ensure a reduction in the type of adverse event being investigated (Patel & Boster, 2025; Wu et al., 2008). Since its original development, RCA has been used as an investigative process spanning a range of fields, including healthcare.

Following serious incidents in mental health, the RCA method is used to gain understanding into the precipitants behind the event, and whether any aspects of the assessment or management of the event that could have been done differently to prevent it. However, some evidence suggests that RCA may have limited impact on improving service delivery (Deshpande et al., 2023; Kellogg et al., 2017; Vrklevski et al., 2018) or encouraging innovative service change (Fröding et al., 2024) in a mental healthcare setting. Interestingly, research by Vrklevski et al. (2018) found that in a review of 27 RCA reports (80% of which involved patient suicide), 70% found no root cause and 38% of the reports resulted in no recommendation for system improvements. Further, the recommendations coming from the RCAs were often seen as being disconnected from the issue, and often far beyond the scope of what mental health services are able to implement (Vrklevksi et al., 2018).

Studies show that even rigorous processes like RCAs can fail to account for all factors involved in patient suicide (Deshpande et al., 2023; Haylor et al., 2024). Causes of suicide, especially with patients living in the community, often involve a unique and complex interplay between people, services, and environmental factors (Deshpande et al., 2023; Haylor et al., 2024). Although there are specific cases where engaging in a root cause analysis may yield important findings and recommendations, in community services, investigations which look for ‘a root cause’ are likely to fail (Vrklevski et al., 2018). It is also clear that the RCA process on its own may not be sufficient to address staff trauma, service development or systems improvement.

In order to overcome some of the limitations of the RCA, a *serious incident review panel* (SIRP) process has been trialled in Australia. This method integrates the principles of restorative justice and recovery-focused mental health support, by seeking input from multiple sources, and using a dialogical approach to knowledge gathering. Through this process, the SIRP aims to empower clinicians and services to both identify possible improvements and acknowledge when things are beyond any one person’s control. Such methods move away from causal analysis, towards a shared knowledge-building of the multiple factors involved and how to best move forward and integrate the new knowledge to improve practice.

## Implementing the Serious Incident Review Panel (SIRP) Process

The SIRP process discussed in this paper has been implemented in a community-based, publicly funded child and youth mental health service over the past 10 years. The service currently has more than 1300 registered clients, and more than 150 staff who provide outpatient care. The multi-disciplinary care teams at the service include psychiatry, psychology, allied health, psychosocial and lived experience expertise. Serious incidents requiring SIRP review are identified by either the management committee or the clinical leaders working across the program. These serious incidents can involve client suicides or near suicides, homicides, or other serious incidents such as near fatal self-harm, severe and repeated self-injuries, complex presentation and difficulties with engagement. The SIRP acknowledges the importance of accountability and learning in systems of care, while also implementing dialogically informed principles aligned with restorative justice and meaning-making. The focus of the review is to assess the quality of care provided to the young person, involving the clinicians who provided care to the client and where possible, the family. The primary aim of the SIRP is not to assign fault to any one party or event, but to analyse the situation as a whole and identify areas of improvement, prioritising compassion, kindness, and the improvement of therapeutic alliances between clinicians and clients. A secondary aim of the SIRP is to support staff to manage emotional distress and remain confident in their ongoing practice. To achieve these aims and ensure the psychological safety of those involved, the SIRP draws on “learning circles” principles, which involve collaborative and reflective assessments where people coming from multiple perspectives can learn from each other (Grealish et al., 2019), while removing the complexity of clinical hierarchy. The SIRP encourages open and transparent discussion, resulting in increased empathy, awareness, reflective thinking, and allowing respectful relationships to be built (Kehoe et al., 2018).

The structure of the SIRP involves a formally constituted panel of 5–6 senior staff members, including both clinical and lived experience staff, with the clinical treating team and any others involved in the care of the young person. This panel participates in every SIRP and provides a space to reflect on what was challenging about the case, and how things could be thought about differently. The panel approaches the meeting with an understanding that the clinical team will have the best sense of the care that has been provided to the young person and their family, rather than approaching the review from the perspective of being the experts. The process of undertaking the SIRP is outlined in Table 1.

**Table 1:** Stages of the SIRP Process

1 – Before the panel meets	**The treating team decide with the panel about who should be involved in the meeting.**	No prior planning or preparation is required.
2 – On the day	**Nominated panel member to have a conversation together with treating team clinicians; panel members listen to the conversation.**	Can you tell us about your experience working with the case? There is an emphasis on openness and getting everyone’s perspective The panel member pays particular attention to the worries and concerns of the team
3	**Panel members to form a reflecting team and talk with each other; One member of the reflecting team to take notes during the reflection**	What did you notice listening to the conversation? What seemed to be most concerning for the team? What struck you as the strengths of the case and the work? Did you have any thoughts about how to strengthen the care, in considering the team’s greatest concerns?
4	**Treating team clinicians to speak with each other in response to the reflection**	What is your response to the panel’s reflection? Any thoughts about what might happen next?
5	**All the group talk together about the outcomes of the discussion**	Open discussion between all members about the review and any possible outcomes.
6	**Nominated panel member to draft a report to be circulated to the team and panel members for comment**	
7	**Final report circulated**	

## Research Aim

The aim of the present study is a real-world examination designed to understand the experiences of individuals who have taken part in a SIRP, both panel members and members of the treating team. The study aimed to understand their views on what works well with the current model, what could be improved, and how the use of the SIRP process has influenced the service more broadly.

## Method

### Participants

Participants in the present study were employees at a youth-focussed mental health service in Melbourne, Australia, who had participated in one or more serious incident review panel meetings, ranging from one (for a team member) to approximately 50 (for the chair of the panel). Eleven staff members took part in the study, holding a range of different professional roles within the service, including psychiatrists, occupational therapists, nurses, social workers, and family lived experience peer workers. Participants had varied lengths of service, ranging from being in their first year of practice, to more than 20 years working in mental health care. Some participants, but not all, had also previously taken part in a Root Cause Analysis following a serious incident. Participants included senior managers as well as people working in multi-disciplinary teams. Semi-structured interviews were conducted face-to-face or using videoconferencing with interviews lasting an average of 24 min and 24 s (range 13:22–47:26). All interviews were recorded, and transcribed verbatim, before all transcripts were checked for accuracy.

### Thematic Analysis

A reflexive thematic analysis was conducted using the transcripts of the interviews, based on the stages of reflexive thematic analysis outlined by Braun and Clarke (2019; see Table 2 for the steps taken in the current study). This involved an open acknowledgment of the experiences and perspectives brought from each of the researchers, and how these specific views may impact the coding process. Researcher reflexivity was a priority in this process and was continually discussed at each meeting of the research coding team. The three researchers (NA, RW, and LH) involved in the thematic analysis process included an academic researcher, a psychiatrist, and a psychologist. Two of the three researchers were experienced in the thematic analysis process.

**Table 2:** Stages of thematic analysis

Stages	
Stage 1 – Familiarising yourself with the fata	NA, RW, and LH met to discuss the project and get a copy of all the transcripts. The aims of the study were discussed, as well the stages of thematic analysis and how to begin the analysis process. Each team member then read through all the transcripts individually to get familiarised with the data. During this stage, any initial points of interest were identified as possibly being important to the analysis.
Stage 2 – Generating initial codes	The group discussed initial thoughts regarding similarities among the data and potential points of meaning. The coding process was also discussed, with an agreement from each person to re-read the transcripts and start grouping the data based on shared significance using a thematic table. These sections outlined in thematic table were initially placed in sections according to the questions asked in the interviews (See appendix A for the interview questions).
Stage 3 – Generating initial themes	The group met twice during this stage to discuss their initial themes and codes and the process by which they came up with these. Initial themes were compared and discussed and quotes from the data were identified to help unpack the themes. During this process the team discussed their subjective views and perspectives and how these may have affected the data analysis. For example, two of the researchers had extensive experience in qualitative coding, while another was wholly unfamiliar with the process. Any differences in the coding process were identified, and the format of a thematic table was agreed upon.
Stage 4 – Reviewing themes	The team reviewed their identified themes and explored the deeper meaning underlying some of the content of the themes/quotes. The researchers confirmed the identified themes and then collapsed or removed some themes based on the extent to which they held meaning, related to other themes, and addressed the aims of the study.
Stage 5 – Defining and naming themes	Another meeting was held to further discuss the meaning of each theme, and the names of the themes were agreed upon. This process involved open discussion of each individual’s views about the meaning of the themes. The agreed names and definitions were discussed in relation to addressing the aims of the study, while also making sure these were accurately capturing what was communicated in the data
Stage 6 – Writing up	Finally, the researchers were all tasked with contributing to a working document of the final report to introduce, describe, and discuss the findings. Many quotes were originally included in the write up of the results, and during this stage, the quotes were reduced to provide more concise examples of the themes.

## Results

As a result of this in-depth reflexive analysis process, nine main themes were identified, which have been organised under three overarching categories. The categories are: the process of the SIRP; the impact of the SIRP on the service; and the challenges and suggestions for improving the delivery of the SIRP. The identified themes in each category are outlined in Table 3.

**Table 3:** Final Themes from Thematic Analysis

Category	Theme	Number of participants who mentioned this
The process of the SIRP	Multiple perspectives in the room	10
	Free flowing process	4
	Supportive process	9
Impact on Service	Non-defensive, recovery-focussed care	7
	Increased staff well-being	4
Challenges and future recommendations	Changes to the process and preparation for SIRP	6
	Including the family	9
	• More orientation and knowledge about the SIRP	8
	• Feedback process to the team/service	4

### The Process of the SIRP

Participants identified a number of practices and processes which occurred during the SIRP which they felt were important to it running efficiently, and that positively impacted their experience of taking part. These themes related to dialogical aspects of the panel meeting as well as providing a supportive rather than punitive response.

#### Multiple Perspectives in the Room

When describing what they felt were the important parts of the SIRP, the majority of participants described that they valued having people with different perspectives in the room. Having multiple perspective in the room was seen as providing a more holistic feel to the process, which stretched beyond making the medical perspective the focus of the panel and encouraged understanding from a variety of different viewpoints.

*I think having those varied kind of professionals helps you think a bit more broadly about the case rather than just through medical point of view or just from the clinical case management point of view. [P4]*

The unique perspectives of the lived experience expert was also mentioned as being an important feature of the SIRP:

*I think there is a good representation of clinicians and lived experience. I think it is really important to have lived experienced staff* *... I think that’s a very important feature of the SIRP. [P3]*

Having multiple perspectives from across the organisation in the review, working collaboratively helped the process to be more egalitarian to flatten the hierarchy within the service:

*It’s not hierarchical in terms of*,* it’s got a group of people that are coming from all different places in the organisation*,* and that it’s with the people who were directly involved in the care. [P10]*

The non-hierarchical structure appeared to create a psychologically safer space for the people involved, however it was also mentioned that having people higher up in the organisation be part of the process provided comfort.

*The consultant* [senior psychiatrist] *has been part and parcel of much of the work and there was a sense of safety around her being there. [P9]*

For this individual, having the senior doctor be part of the process was important in providing that safe environment, and to feel that senior staff were supportive and ‘had their back’ through this challenging time.

#### Free Flowing Process

Another aspect of the process that was described as beneficial was that communication felt organic and free flowing. This environment meant that people could talk at their own pace without feeling under pressure to answer a set of predetermined questions:

*…most of the panel were coming in and responding to the information that was presented during the interview process rather than having some preconceived ideas or questions or concepts to bring to the thinking. [P6]*

This approach could allow the focus of the panel to evolve more organically, going in multiple directions:

*[It] allows a bit more sort of free association of what is transpiring. [P8]*

#### Supportive Process

Participants in the SIRP felt that it was a personally supportive process. In contrast to previous experiences, individuals felt supported by the process and felt that it was not punitive towards them:

*what the SIRP probably allows people to do is is to feel backed up by the service… I think it also helps staff feel quite cared for. [P6]*

The dialogic approach extended beyond an examination of areas for improvement, and included reflecting on the positive aspects of the case management which, again, added to a sense of feeling supported. Especially being held through the difficult process of a young person dying while under the care of the service:

*I found it a really supportive process to go through and to really examine the work that we did and to… think… maybe there was some recommendations or some learnings*,* but also …. I think it was quite powerful to hear that we had done a really good job. [P4]*

For staff going through an incident review, it was important for them to feel validated and that their work was appreciated.

*…it’s sort of about feeling validated or feeling supported in the work that we did. [P5]*

*I feel like it’s very validating for that staff member to feel that level of support from many different levels. [P4]*

This acknowledgement and validation resulted in staff feeling grateful for participating and that process was a positive experience overall.

w*hen I became involved in the SIRP*,* I was a little bit frightened that I wouldn’t serve the passed away person*,* the clinical teams*,* and the family well*,* but it was such a great group*,* professional group that it was an absolute honour to be involved in the SIRP the way we did the process was so respectful and communicative. [P7]*

This validation was felt by panel members as well as case managers, who were able to feel that they were actively involved in supporting staff through difficult experiences.

*I feel very honoured really*,* to be sitting on the SIRP and be*,* you know*,* involved in*,* and thinking about how we support our staff and the families where there has been loss. [P8]*

### Impact on Service

The second category of themes related to broader impact on the service. Many participants reflected that the SIRP impacted the service at a systemic level. These service-level changes have been grouped into the themes of: n*on-defensive*,* recovery-focussed care*; and *increased staff well-being*.

#### Non-Defensive, Recovery-Focussed Care

Participants felt that the SIRP process improved the capacity of staff to work in a manner that was non-defensive and not overly governed by risk and self-protection, and could encourage taking a person-centred approach:

*It doesn’t negatively impact our ability to carry and manage risk for other clients*,* because I think when it’s not done well*,* it can have a negative impact of increasing more risk adverse practises*,* more restrictive measures and being less collaborative with clients and families to be able to tolerate risk*,* to get a better clinical outcome. [P3]*

A number of individuals discussed feeling supported to be courageous in their work and moving away from the defensive posture required when blame is apportioned:

*I think with incident reviews you know it*,* it can really change how the service works and I think incident reviews that feel blaming and feel like they’re trying to find what was wrong actually can kind of inadvertently create a culture of people practising in a very scared*,* defensive manner. [P6]*

*It really helps us continue to provide a courageous service that is able to meet the needs of those who are seeking help and not slip into lots of risk adversity because of fear of blame and scrutiny. [P10]*

When comparing the SIRP to previous experiences with other incident reviews, individuals also felt there was a stark contrast in the focus of the review, away from investigating the specifics of the incident and towards an examination of the holistic and systemic factors at play.

*I think that the biggest difference is that it’s [RCA] an investigation that’s seeking to understand the cause of the event*,* whereas our process is a retrospective review of the care*,* that’s meant to be looking at instilling strength in the system again. [P1]*

#### Increased Staff Well-Being

Interestingly, beyond the experience of the review being supportive ‘in the room’, individuals felt it was supportive to the organisation and had a positive impact on staff mental health, with the possibility of this having a positive flow on effect for staff retention:

*I think it really minimises the trauma to the organisation following something like a suicide and also… it’s better in terms of staff retention. I think it’s better in terms of staff wellbeing. [P3]*

*It helps people feel able to stay in it*,* especially after some bad things happen. So it helps us with our culture. It helps us reduce the impacts of systemic trauma and individual vicarious trauma. [P10]*

### Challenges and Future Recommendations

The final set of themes centred around participants’ views about the *challenges associated with the SIRP*, and the *future directions for an improved process*.

#### Challenges Associated with the SIRP

Despite participants indicating that taking part in the SIRP was supportive and validated their practice, some people disclosed that they entered the SIRP with some fear and apprehension of what to expect. Indeed, some individuals had been wholly unaware of the SIRP purpose and process before attending.

*I don’t think the majority of the staff actually know what the SIRP is or what it’s for… Having better communication*,* written materials or something could be helpful so that staff [who] were new to the process feel less anxious*,* going in. [P3]*

This sentiment was also echoed by a staff member who felt that greater preparation and information about the process might help people alleviate existing worries that may have been shaped by previous experiences with incident reviews:

*I think that people will naturally believe that a serious incident is always going to be looking at blaming or you know… you’re almost put on trial to explain what you’ve done. So I think maybe training staff around what a SIRP actually is… makes sense. [P11]*

There was also the suggestion that following the SIRP, in addition to increasing transparency and knowledge about the SIRP process, providing feedback to other team members following the process may be beneficial.

*Maybe it might be good to have someone come to a team meeting and kind of feedback about it from the panel. [P4]*

### Recommendations for the Future

Beyond the need for better preparation, participants discussed the possibility of including the family in future iterations of the SIRP, recognising both that the family’s voice was important and necessary, while acknowledging the need to be respectful and aware of families’ preferences, especially where there has been a death. While the service is committed to transparency and open communication, there remain challenges in including the voice of the family, recognising that not all families will want to be involved, that staff may feel awkward or constrained in their responses and that when the young person is still alive, balancing the needs of the young person against the needs and wants of the family may be particularly vexed. One option is to create a two-step process, as one staff member described:

*To be honest and not always do we give the family the copy of the report now*,* so it feels a bit uneasy me saying that because I’m not sure that’s right. And I think we probably should change that practice… The last SIRP we did*,* we did one of our panel members went and interviewed the family separately to the process and brought the information back. That seemed like a good compromise to me and I think we should try doing that more often. [P1]*

Another participant described a SIRP where the young person had been involved, and the transformative effect it had had on her care

*There was one where family members were interviewed with a girl who was very unwell with an eating disorder*,* and it sort of helped change the whole dynamic*,* power dynamic between the sort of conflictual relationship for the family and the service to a more collaborative one.*

## Discussion

The aim of the present study was to explore the views and experiences of individuals who had taken part in one or more serious incident review panels, and to consider whether the operation of the SIRP met the needs of the service, the staff members and the young people and families involved. The themes among the data indicated that, overall, participants found taking part in the SIRP positive and supportive and felt that the SIRP process contributed to a braver, person-centred, and supportive service. Participants also felt there was room for improvement in implementation, especially in relation to the preparation before taking part and the need for more information about the SIRP to be provided across the service.

Overall, participants reported that the SIRP process had been supportive for them, especially when dealing with challenging impacts of the incident. This is an especially important finding and addresses many of the documented issues aligned with investigating serious incidents, which can sometimes feel punitive and like the focus is on finding fault in an organisation and/or their staff (Croft et al., 2023; Vrklevski et al., 2018). In contrast, participation in the SIRP was seen to be a mostly positive process, and that even though some participants were initially apprehensive, they left feeling supported, both by others in the room and from the organisation, represented by the senior staff present. Potentially, when the approach taken is proactive and engaging rather than rule-based, it can foster intra-organisational relationships with higher levels of respect and empathy (Kehoe et al. 2018).

The supportive nature of the SIRP process also appeared to be facilitated by the inclusion of multiple perspectives in the process. This was consistently highlighted by the participants and supports recent research suggesting that this is important for building understanding and transparency (Kok et al., 2022) and reducing epistemic injustice (Grim et al., 2019).

Another widely discussed feature of the SIRP was the free-flowing nature of the process itself and that it did not run according to a pre-determined checklist or need to produce a pre-structured report. This theme is congruent with the dialogically informed nature of the SIRP, where the death or other serious incident is discussed openly, with the aim of respectful knowledge building and meaning-making, and a focus on future improvements rather than retrospective blame.

Interestingly, the interviewees viewed the impact of the SIRP as stretching beyond the review itself and into the service more broadly, as staff felt the process encouraged the continuation of brave clinical practice within the service. This finding is significant given prior research has shown that incidents like client suicides can lead to more defensive practice (Croft et al., 2023; Sandford et al., 2020) at the cost of more relational and holistic care (Kelleher & Campbell, 2011). Despite the well-documented impact of client suicides on clinicians, where they may feel a sense of responsibility for the death or increased professional doubt (Haylor et al., 2024; Pratt & Jachna, 2015; Sandford et al., 2020), the present results highlight the potential moderating impact of providing adequate support following these incidents (Sandford et al., 2020).

Despite overall positive experiences with the SIRP, staff felt there was not adequate preparation for participants and that it would be beneficial if there was formal feedback regarding the outcomes of the SIRP across the organisation, so that teams involved with incident could understand the process and what was learnt. This finding has also been suggested in relation to the more formal RCA process where participants felt that better communication about the outcomes of the process were needed (Vrklevski et al., 2018). Including more feedback about serious incident reviews may provide a beneficial ‘feedback loop’ where both the teams involved, and the service more broadly can benefit from the findings and learnings from the review, which can then positively impact future work practices. Future iterations of the SIRP were also discussed as potentially benefitting from having more involvement from families. This sentiment from staff has also been supported in recent research, that highlights the importance of including family members to provide a fuller picture on what was happening for the person involved (Fröding et al., 2021; Kok et al., 2022). Recent research by Wiig et al. (2021a) also showed that for those investigating adverse events, involving next of kin could provide a better understanding of the case overall and improve the quality of the investigation. The participation by family and next of kin in the process was also therapeutic for the families themselves knowing that their involvement could contribute positively to the investigation (Wiig et al. 2021b). Ramsey et al. (2025) also suggest the need for investigations of serious incidents to consider the needs of those affected, such as families, and to ensure patients and their relatives are meaningfully involved in the process where possible. However, involving families in reviews such as the SIRP also need to consider client specificity and honour the unique needs of those involved, including if they do not wish to participate.

### Implications

There are a number of implications from the findings of the present study for the ways in which mental health services investigate serious incidents. As there have been numerous critiques of serious incident investigation processes as being disconnected those who provide care (Vrklevksi et al., 2018) and potentially being harmful to those involved (Ramsey et al., 2025), the present findings suggest there are ways of investigating serious incidents that may help to ameliorate some of these adverse effects. By running a process like the SIRP alongside any mandated incident investigations such as RCAs, it can provide a complementary process that is supportive to staff alongside processes that often do not have staff well-being as a major consideration. The findings suggest that implementing a SIRP process to investigate serious incidents can lead to positive change, be a supportive process for the clinicians, and can positively impact the wider service. The findings further suggest that mental health services can run their own investigations that involve people directly involved with the case and use their insights to learn about what might have contributed to the incident, and how to best mitigate risks in the future. What appears to be crucial in this process for services is for senior staff to be actively involved in the process in a supportive way, where there is an open dialogical process, which facilitates the ongoing courageous practice of those involved.

### Limitations and Future Research

Despite the interesting findings in the present study, there are a number of limitations present. First, the sample in the present study was relatively small (11) and all came from a single mental health service which limits the generalisability of the findings to other services. In the present study, the mental health service strongly values the voice of lived experience, and aims to provide dialogically informed, person-centred, recovery focused care to clients. Therefore, it may be well-suited to a process like the SIRP that is congruent with these values. For services that may have a stronger focus on the medical model, rather than a holistic recovery focus, a process like the SIRP may not be as easily implemented or be aligned with the core values of the service. Another potential limitation of the SIRP is that it did not measure any outcomes arising from the process, and thus its impact in relation to service change or reduction in similar incidents cannot be ascertained. Future research into processes like the SIRP may benefit from monitoring outcomes and changes that result from the SIRP process to measure what impacts, if any, the process has in changing service provision, better meeting client needs, and improving staff well-being. Furthermore, based on recent research (Wiig et al. 2021a, b) and the present findings, future iterations of the SIRP may benefit from thoughtfully choosing to include family members involved with the incident, both to gain further insights, but also to assist in the meaning-making and healing process for all those involved.

## Conclusion

The findings from this study highlight the overall positive experiences of those who take part in SIRPs and indicate the potential benefits for mental health services to reorient the way that serious incidents, such as the death of a client, serious self-harm, persistent aggression, and other challenges are managed in a restorative rather than punitive way. These findings are in line with contemporary literature, which continues to shift the narrative in mental health care away from rigid, linear models of causative explanations towards respectful, person-centred and recovery-oriented care. Despite limitations, the present findings have implications for other services who may wish to implement new and complementary ways of dealing with serious incidents that are based in restorative justice, are dialogically informed, supportive, and involve collaborative learning from the past to improve the future.

## Data Availability

No datasets were generated or analysed during the current study.

## Appendix – Interview Questions

**Semi structured interview schedule – SIRP**.

1. What is your role in the SIRP?
2. How long have you been in this role/How many times have you participated in a panel?
3. Have you been involved in an RCA prior to the introduction of the SIRP?
4. If so, could you describe how the SIRP differs from the RCA?
5. Did you receive any training prior to participating in SIRP?
  - If yes, was it useful?
  - If no, what training would have been useful?
6. What are the things which work well with the SIRP?
7. What are the things which don’t work well/What things could be done better?
8. What are the benefits for staff who participate in SIRP?
9. What are the benefits for the organistion?
10. What are the benefits for young people/families (if any)?
11. What are the difficulties for staff who participate in SIRP?
12. What are the difficulties for the organistion?
13. What are the difficulties for young people/families (if any)?
14. Do you think the SIRP should continue in it’s current form?
  - If not, what should change?
15. Are there any other comments about the SIRP?
